# Surgical management of symptomatic hemangioma of the geniculate ganglion: fascicular-sparing resection or grafting?

**DOI:** 10.1007/s10143-023-02029-w

**Published:** 2023-05-15

**Authors:** Alice Giotta Lucifero, Sabino Luzzi, Jessica Rabski, David Meredith, Paulo Abdo do Seixo Kadri, Ossama Al-Mefty

**Affiliations:** 1 Department of Clinical-Surgical, Diagnostic and Pediatric Sciences, Neurosurgery Unit, University of Pavia, Pavia, Italy; 2 Department of Brain and Behavioral Sciences, University of Pavia, Pavia, Italy; 3 Department of Surgical Sciences, Neurosurgery Unit, Fondazione IRCCS Policlinico San Matteo, Pavia, Italy; 4 Brigham and Women’s Hospital, Harvard Medical School, Boston, MA USA; 5 Department of Pathology, Brigham and Women’s Hospital and Harvard Medical School, Boston, MA USA; 6 Medical School, Federal University of Mato Grosso Do Sul, Mato Grosso Do Sul, Campo Grande, Brazil

**Keywords:** Facial nerve function, Facial nerve hemangioma, Fascicular-sparing technique, Grafting, Hemangioma of the geniculate ganglion, Neuromonitoring

## Abstract

Geniculate ganglion hemangioma (GGH) is rarely presented in the neurosurgical literature. It extends extradurally on the middle fossa floor and displaces the intratemporal part of the facial nerve. Surgical treatment is advisable at early symptoms. Proposed techniques include fascicular-sparing resection or nerve interruption with grafting. No definitive conclusions exist about the superiority of a certain technique in preserving facial nerve integrity and function. Through the description of a surgically managed symptomatic GGH, we herein discuss literature data about the surgical results of fascicular-sparing resection versus grafting. A PRISMA-based literature search was performed on the PubMed database. Only articles in English and published since 1990 were selected and furtherly filtered based on the best relevance. Statistical comparisons were performed with ANOVA. One hundred sixteen GGHs were collected, 56 were treated by fascicular-sparing resection, and 60 were treated by grafting. The facial function was improved, or unchanged, in 53 patients of the fascicular-sparing group and 30 patients of the grafting one. Sixty-five patients achieved a good (House–Brackmann (HB) grade III) postoperative facial outcome, of which 47 and 18 belonged to the fascicular-sparing and grafting group, respectively. Greater efficacy of the fascicular-sparing technique in the achievement of a better facial outcome was found (*p* = 0.0014; *p* = 0.0022). A surgical resection at the earliest symptoms is critical to preserve the facial nerve function in GGHs. Fascicular-sparing resection should be pursued in symptomatic cases with residual facial function (I–III HB). Conversely, grafting has a rationale for higher HB grades (V–VI). Broader studies are required to confirm these findings and turn them into new therapeutic perspectives.

## Introduction 

Geniculate ganglion hemangiomas (GGH) are rare, benign, slow-flow vascular lesions accounting for 0.7% of intratemporal tumors [1–3]. They grow from the vascular plexus around the geniculate ganglion and frequently extend into the facial nerve’s internal auditory canal, labyrinthine, and tympanic segment [4, 5]. The first case of GGH was reported by Pulec in 1969 [6]. They described a vascular neoplasm within the temporal bone, liable for significant facial dysfunction [6]. Since their early stages of growth, GGHs have a symptomatologic onset characterized by a sudden or progressive peripheral facial nerve palsy or might be associated with hemifacial spasm. The subsequent involvement of the auditory nerve and the erosion of the cochlea and ossicular chain result in conductive or sensorineural hearing loss [7, 8].

The true incidence of GGHs is underreported as it is frequently misdiagnosed since epidemiology data mainly came from a few case reports and brief reviews [9–15]. Although advances in neuroimaging techniques enabled the identification of distinctive radiological features, an initial differential diagnosis is still challenging [16, 17]. GGHs are often mistaken for schwannomas of the facial nerve or middle fossa meningiomas, leading to erroneous management. The therapeutic choice is affected by several factors as the patient’s age, symptoms, deficits, tumor features, and extension.

Despite a wait-and-see approach which is also considered a valuable option in selected patients, literature data reported surgery as the best treatment option for symptomatic GGHs at the earliest sign [1, 6].

During surgery, the main challenge is to dissect the facial nerve from the tumor, save the fibers, and preserve its function. Two different surgical techniques have been reported: the fascicular-sparing resection of the tumor and the facial nerve interruption with grafting.

Herein, we reported a systematic literature review about the surgical management of GGH, focusing on the comparison between the results of the fascicular-sparing resection versus grafting. The case of a 38-year-old symptomatic patient harboring a GGH and surgically managed with the facial-sparing technique is also discussed.

## Methods

A comprehensive online systematic review was performed in accordance with the Preferred Reporting Items for Systematic Reviews and Meta-Analyses (PRISMA) guidelines [18]. We queried the PubMed/Medline (https://pubmed.ncbi.nlm.nih.gov, accessed on 13 December 2022) electronic database using combinations of the following search terms and words text: “geniculate ganglion hemangioma”, “ganglional hemangioma”, “hemangioma of the facial nerve”, “facial hemangioma”, and “intratemporal hemangioma”.

Only records regarding the surgical management of GGHs, written in English or translated and published since 1990, were assessed for eligibility. Reviews, editorials, comments, and articles, including non-surgical treatments, were excluded. Results were further sorted based on their relevance from titles and abstracts. The data extraction protocol recorded the following information: authors’ names, year of publication, demographics, clinical data, and surgical techniques.

### Outcome analysis

The patients were arranged into two groups based on the surgical technique: the fascicular-sparing resection or nerve interruption followed by grafting.

Preoperative facial dysfunction was assessed by means of the House–Brackmann (HB) grading. Grades I–II were reported as a single group having mild facial dysfunction, normal tone, and symmetry at rest. Grade III consisted of a moderate deficit, weakness, synkinesis, and complete eye closure maintained with effort. In grade IV, the patient had a moderately severe dysfunction with weakness, disfiguring asymmetry, and incomplete eye closure. Grades V–VI, considered together, referred to a severe deficit, barely perceptible motion, or total paralysis. The association between the groups (I–II and V–VI) within the grading system comes from the assumption that eye closure was considered the most critical factor affecting the patient’s quality of life, as stressed in the literature.

The overall postoperative facial outcome was reported as improved, unchanged, or worsened.

The repeated-measures analysis of variance (ANOVA) was applied to compare patients with different HB grades within each group. The rate of improved/unchanged facial outcome was evaluated. The achieving of a good facial outcome, intended as HB grade ≤ III, was also estimated. The reason for having set HB grade III as a cut-off for a good facial outcome lies in the fact that it is the higher grade with a preserved eye closure function. Only patients admitted with full or partially preserved facial function (HB I–IV) were included in the statistical analysis. A *p-*value < 0.05 was assumed as statistical. Prism 5 (GraphPad Software, Inc.) software was used for the analyses.

## Results

### Literature volume

**Fig. 1:**
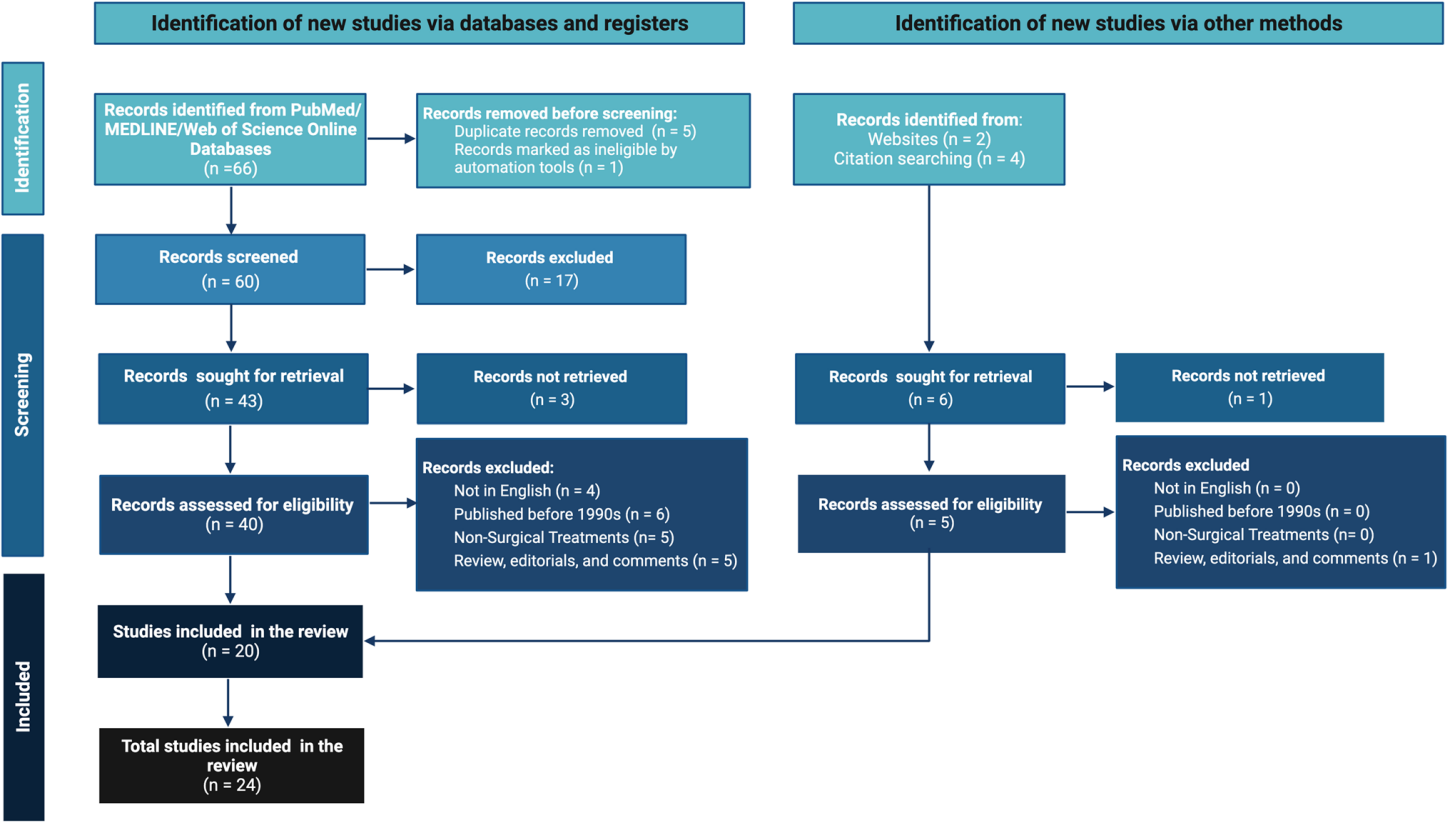
PRISMA flow-chart

The literature search returned a total of 66 records. After the removal of duplicates and screening, 20 articles were assessed for eligibility. Implementation of the exclusion criteria selected 24 articles for the review. Figure 1 presents the PRISMA flow chart for the literature selection process (Fig. 1).

Data collected from the literature about the surgical treatment for GGHs are summarized in Table 1.

**Table 1:** Literature data of surgical management of geniculate ganglion hemangioma

#	Author, year	No. of pts	Age (average)	Sex (No. of pts)	Site (No. of pts)	Side (No. of pts)	Presenting symptoms (No. of pts)	Preoperative HB (No. of pts)	Postoperative HB (No. of pts)	Preoperative hearing (No. of pts)	Postoperative hearing (No. of pts)	Surgical approach (No. of pts)	Graft (No. of pts)
1	Gavilàn, 1990	1	38	M	GG	L	Facial paralysis	VI	VI	Dead ear	Unchanged	MCF	None
2	Balkany, 1991	1	24	M	GG	R	Facial paralysis	VI	III	Preserved	Unchanged	MCF + TM	None
3	Fish, 1992	3	53	F (2) M (1)	GG	NA	Facial palsy (3)	IV (2) V (1)	II (1) III (2)	CHL (1)	Recovered	MCF + TM (3)	GAN (2) GSN (1)
4	Martin, 1992	1	53	F	GG + LS + TS	R	Facial palsy	V	V	Preserved	Unchanged	MCF + TM	CP
5	Bhatia, 1995	20	45	M (12) F (8)	GG	NA	Facial paralysis (12) Facial palsy (8) Hemifacial spasm (1) Tinnitus (1) Otorrhea (1)	I (5) II (4) III (2) IV (4) V (2) VI (3)	I (4) II (1) III (2) IV (2) V (4) VI (5) NA (2)	Preserved (8) Dead ear (7) NA (2) SNH (2) CHL (1)	Preserved (8) Dead ear (8) SNH (2) NA (2)	MCF (5) TM (5) MCF + TM (3) SO (1) TO (1)	None (8) GAN (5) GSN (7)
6	Asaoka, 1997	1	30	F	GG	R	Hemifacial spasm	IV	II	SNH	Recovered	MCF	None
7	Escada, 1997	1	32	M	GG + LS	L	Progressive hearing loss	I	III	CHL; PTA 30 dB	Unchanged	MCF	GSN
8	Friedman, 2002	2	53	M (1) F (1)	GG + TS (1)	R (2)	Hemifacial spasm (1), vertigo (1), nausea (1), tinnitus (1)	I (1) IV (1)	I (1) IV (1)	Preserved (2)	Unchanged	MCF (1) MCF + TM (1)	GAN (1)
9	Piccirillo, 2004	3	47	M (3)	GG (1) GG + LS (1) GG + TS (1)	R (3)	Hemifacial spasm (1) Facial paralysis (3)	III (2) VI (1)	IV (3)	PTA 17 dB (1); 18 dB (1); 60 dB (1)	PTA 22 dB (1); 15 dB (1); 46 dB (1)	MCF + TM (2) MCF (1)	GSN (3)
10	Fierek, 2004	1	6	M	GG + T	L	Recurrent ear infection	I	I	CHL; PTA 5–15 dB	Improved	TM + PORP	None
11	Isaacson, 2005	6	41	M (5) F (1)	GG (5) GG + LS + TS (1)	R (5) L (1)	Facial paralysis (6); tinnitus (1); hearing loss (2); facial weakness (2); headache (1); epiphora (1); blepharospasm (1); hyperkinesis right upper limp (1); facial spasm (1)	II (1) III (1) IV (2) V (1) VI (1)	III (3) IV (2) VI (1)	Hearing loss (3) Absent acoustic reflex (2)	Improved	MCF (5) MCF + TM (1)	GAN (5)
12	Miyashita, 2007	1	47	M	GG	L	Facial palsy	VI	V	Preserved	Unchanged	MCF	GAN
13	Casas-Rodera, 2007	2	30	F (2)	GG	R (1) L (1)	Facial palsy	V (1) VI (1)	II (1) IV (1)	Preserved	Unchanged	MCF	None
14	Capelle, 2008	1	35	M	GG	L	Facial palsy	VI	IV	Preserved	Unchanged	MCF	GSN
15	Sade, 2009	1	45	M	GG	R	Tinnitus	I	I	Progressive loss	Recovered	SO	None
16	Semaan, 2010	15	54	F (11) M (4)	GG	NA	Facial paralysis	I (1) II (5) III (3) IV (2) V (2) VI (2)	I (1) II (6) III (5) IV (2) V (1)	HL (4)	Unchanged (1) Worsened (3)	MCF (13) TA (2)	None (11) GAN (3) GSN (1)
17	Benoit, 2010	7	45	NA	GG	NA	Facial weakness (7) Vertigo (1) Facial twitching (1)	III (1) IV (1) V (1) VI (4)	III (2) IV (5)	Loss (2)	Unchanged	MCF	None
18	Ross, 2013	1	37	F	GG	L	Facial weakness Facial palsy	II	I	Preserved	Unchanged	MCF	None
19	Marchioni, 2014	1	77	M	GG	L	Facial palsy Vertigo Headache	II	III	High-frequency SNH	Recovered	TE + TM	None
20	Wang, 2014	16	43	F (12) M (4)	GG (14) GG + LS (1) GG + TS (1)	NA	Facial paralysis	III (2) IV (4) V (4) VII (6)	I (1) II (2) III (8) IV (2) V (3)	CHL; PTA 40 dB (1) SNH (1) Dead ear (1)	CHL, PTA 25 dB (1) Dead ear (1)	MCF (14) MCF + TM (1) EL (1)	GAN (6) GSN (3)
21	Ma, 2014	12	41	F (8) M (4)	GG	NA	Facial deficits (11) Tinnitus (1)	III (12)	I-II (10) III–IV (2)	CHL; PTA 50 dB (1)	Recovered, PTA 20 dB (1)	MCF	GAN (1) GSN (1)
22	Oldenburg, 2015	8	35	F (5) M (3)	GG	L (7) R (1)	Progressive facial weakness (7) Hemifacial spasm (4) Sudden facial weakness (1) Dizziness (1)	III (2) IV (3) VI (3)	II (1) III (2) IV (1) V (2) VI (6)	SNH (1)	Recovered	MCF + TM (8)	GAN (3) GSN (1)
23	Lahlou, 2016	10	42	NA	GG	NA	Facial palsy	III (1) IV (2) V (4) VI (3)	III (6) IV (2) V (1) NA (1)	CHL; PTA 5 dB (5); 10 dB (3); 11 dB (1); 34 dB (1)	CHL, PTA 7 dB (1); 9 dB (1); 10 dB (3); 14 dB (1); 15 dB (1); 16 dB (1); 41 dB (1); NA (1)	MCF	GAN (9) GSN (1)
24	Bonali, 2019	1	42	NA	GG	L	Facial paralysis	VI	III	SNH; PTA 10 dB	Recovered	TE	None

### Demographics, clinics, and surgical data

Overall, 116 patients underwent surgery for a GGH resection. The age ranged from 6 to 77 years old, with a mean age of 44. Among them, 53% were female.

All the patients presented with facial palsy, associated with hearing loss, hemifacial spasm, tinnitus, vertigo, headache, nausea, ear infection, and epiphora in 87, 15, 4, 3, 2, 1, 1, and 1 case, respectively.

Regarding the intraoperative technique, 56 patients (48%) underwent a fascicular-sparing removal of the tumor, while in 60 cases (52%), the facial nerve was interrupted and repaired by graft. The facial nerve rerouting was performed by means of the greater auricular or sural nerve in 40 and 20 cases, respectively.

**Fig. 2:**
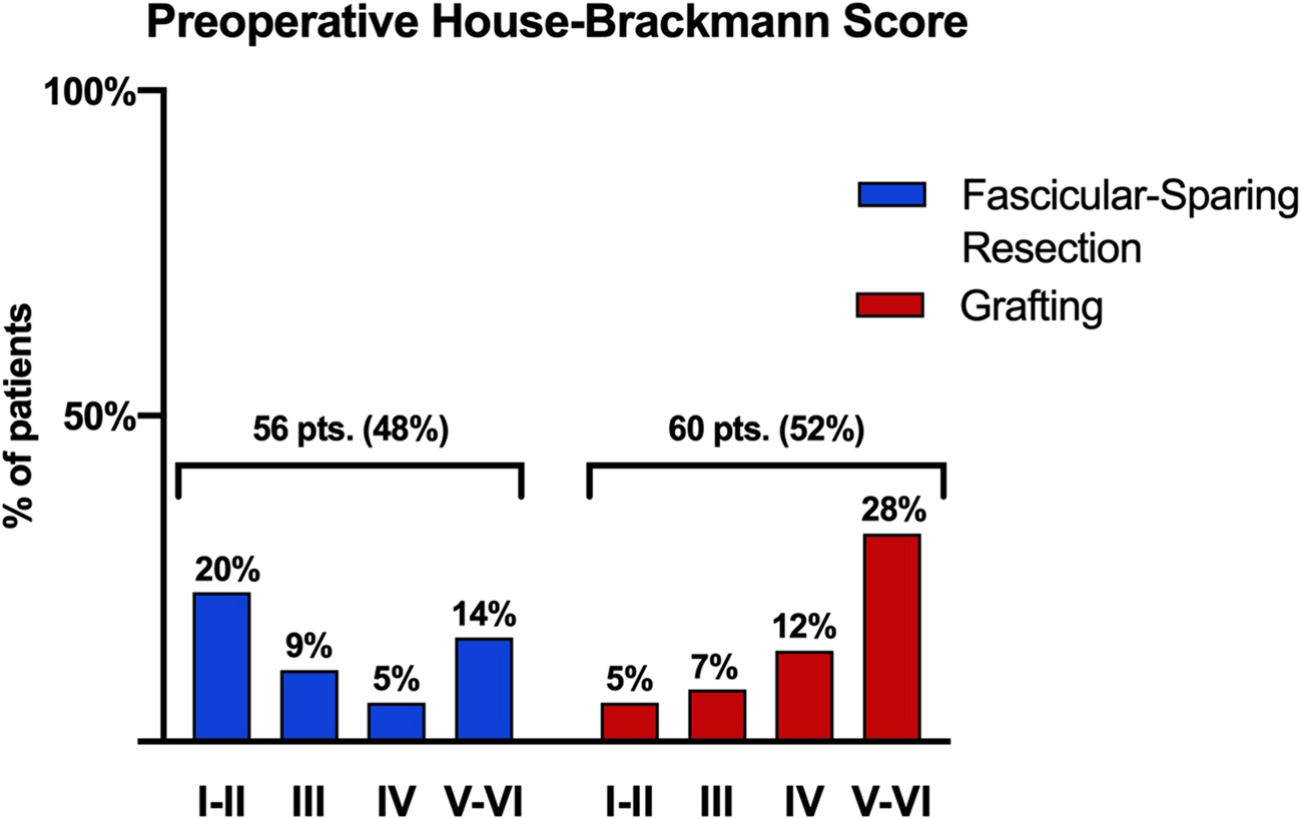
Bar graph showing the preoperative House–Brackmann grade

**Fig. 3:**
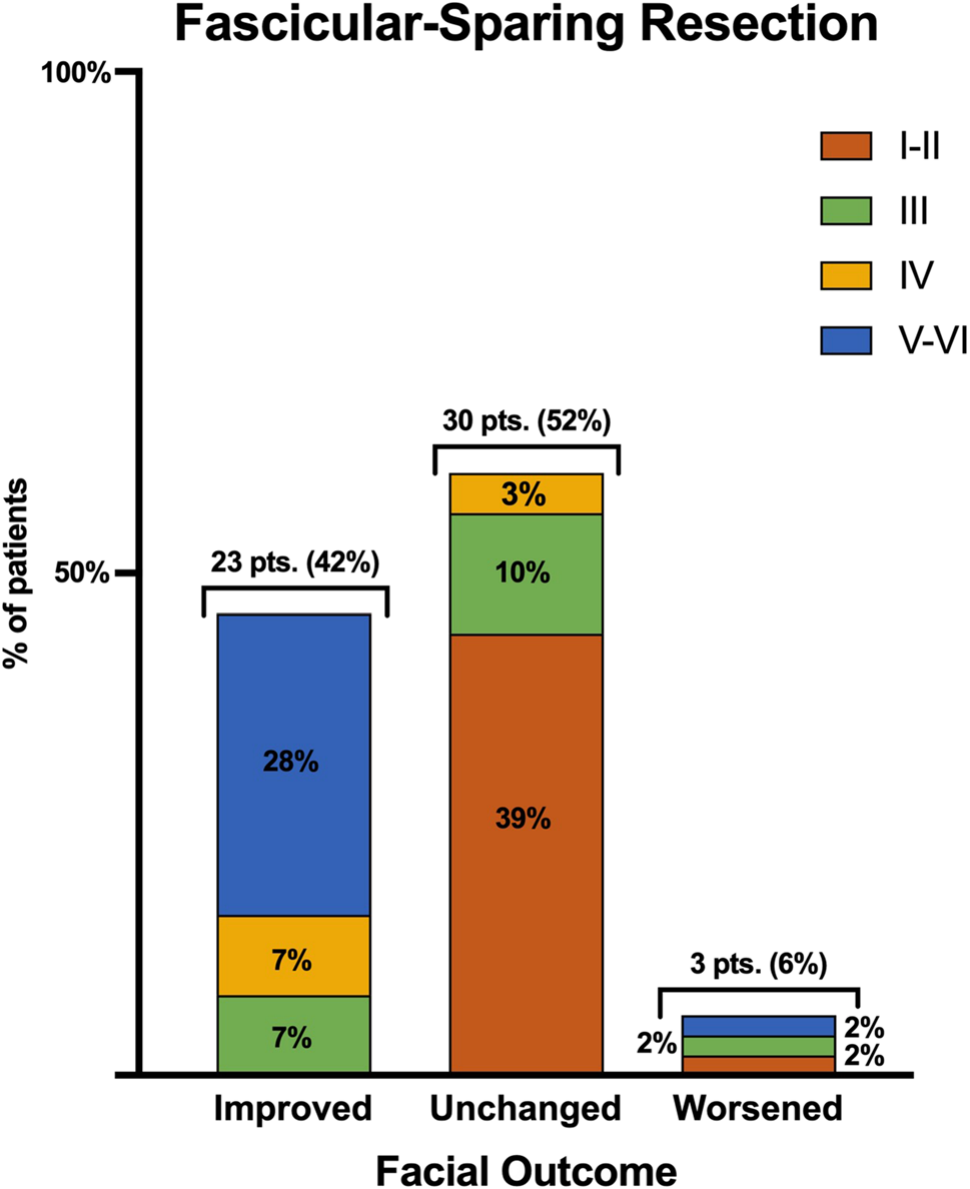
Bar graph reporting the facial outcomes in the fascicular-sparing resection group

**Fig. 4:**
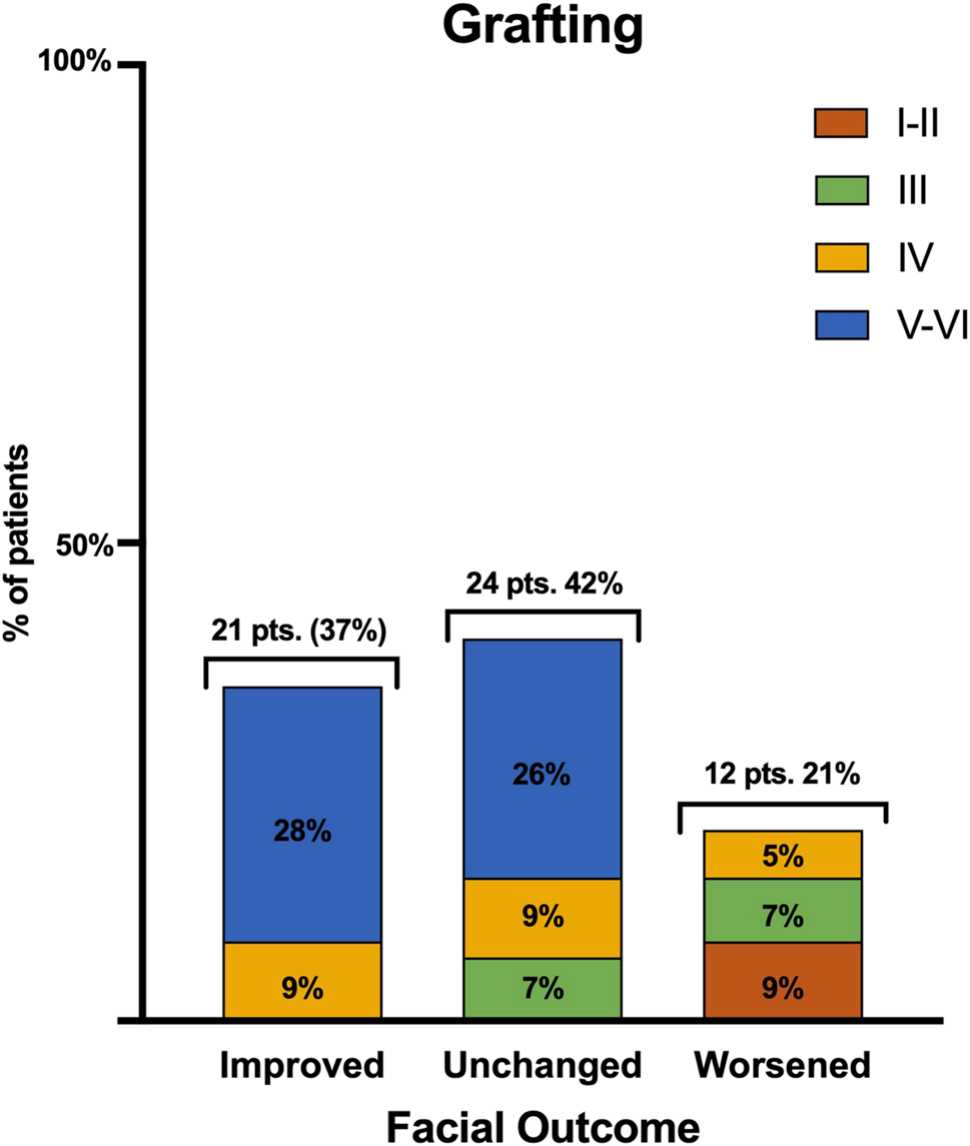
Bar graph describing the facial outcomes in the grafting group

At presentation, in the fascicular-sparing group, an HB grade of I–II, III, IV, and V–VI was reported in 23 (20%), 11 (9%), 6 (5%), and 16 (14%) cases, respectively. Among the patients who underwent grafting, 6 (5%), 8 (7%), 14 (12%), and 32 (28%) patients presented with an HB grades I–II, III, IV, and V–VI, respectively (Fig. 2). Among patients treated through the fascicular-sparing technique, the facial outcomes were improved, unchanged, and worsened in 23 (42%), 30 (52%), and 3 (6%) cases, respectively (Fig. 3). Patients who underwent nerve rerouting achieved an improvement in facial dysfunction in 21 (37%) cases, while facial dysfunction was unchanged in 24 (42%) and worsened in 12 (21%) patients. Three cases were lost at follow-up (Fig. 4).

The two-way ANOVA revealed significant differences between the facial outcomes of the two groups.

When analyzing the facial function of patients with an improved/unchanged outcome, 53 patients (64%) belonged to the fascicular-sparing group and 30 cases (36%) to the grafting one. Among these, those who underwent fascicular-sparing resection of the tumor presented with HB grades I–II, III, IV, and V–VI in 22 (26%), 10 (12%), 6 (7%), and 15 (18%) cases, respectively. Therefore, most patients who gained better results started with an I–III HB. On the other hand, those who underwent grafting had preoperative HB grades III, IV, and V–VI in 4 (5%), 10 (12%), and 16 (19%) cases, respectively.

**Fig. 5:**
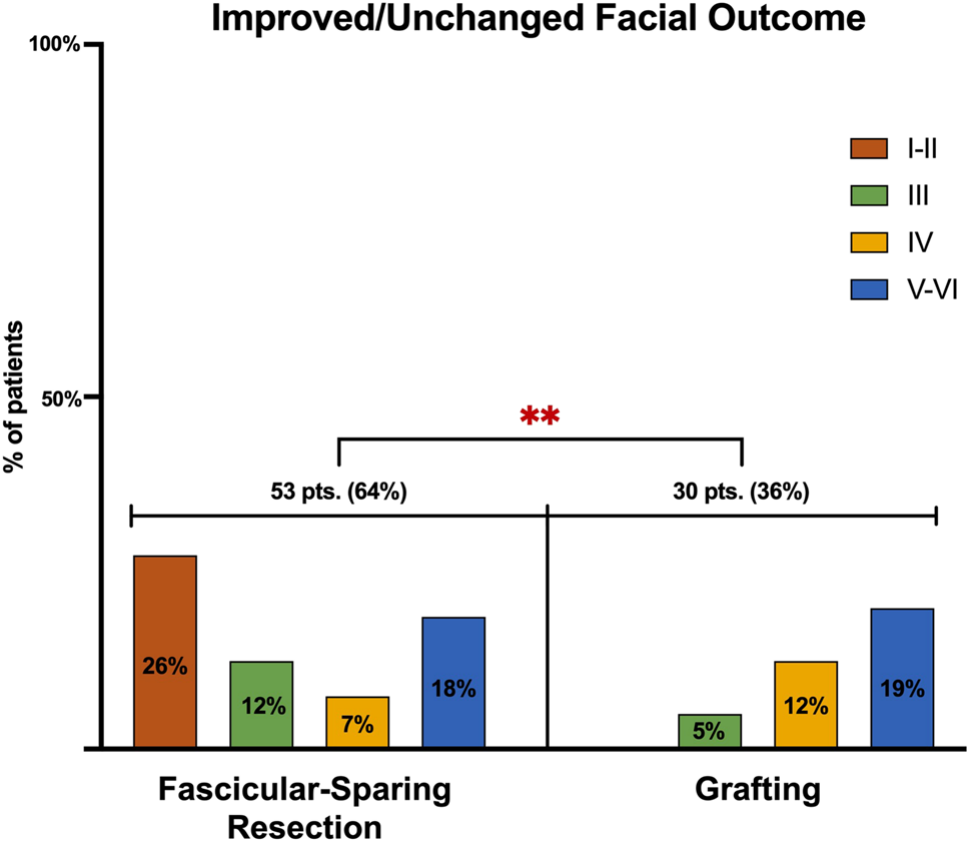
Bar graph showing the statistical comparison between the efficacy of each technique in preserving the facial nerve function in terms of improved/unchanged facial outcomes

Results showed the surgical advantage of the fascicular-sparing technique in reaching an improved/unchanged facial outcome, especially for patients with HB grades I–II and III at admission (*p* = 0.0014) (Fig. 5).

The assessment of patients with an HB grade III who postoperatively recovered a complete eye closure revealed the fascicular-sparing technique’s significant efficacy in achieving a good facial outcome (*p* = 0.0022). Seventy-two percent (47) of patients noted with HB grade III underwent fascicular-preserving removal of the tumor; of these, 34% (22) had an HB I–II.

**Fig. 6:**
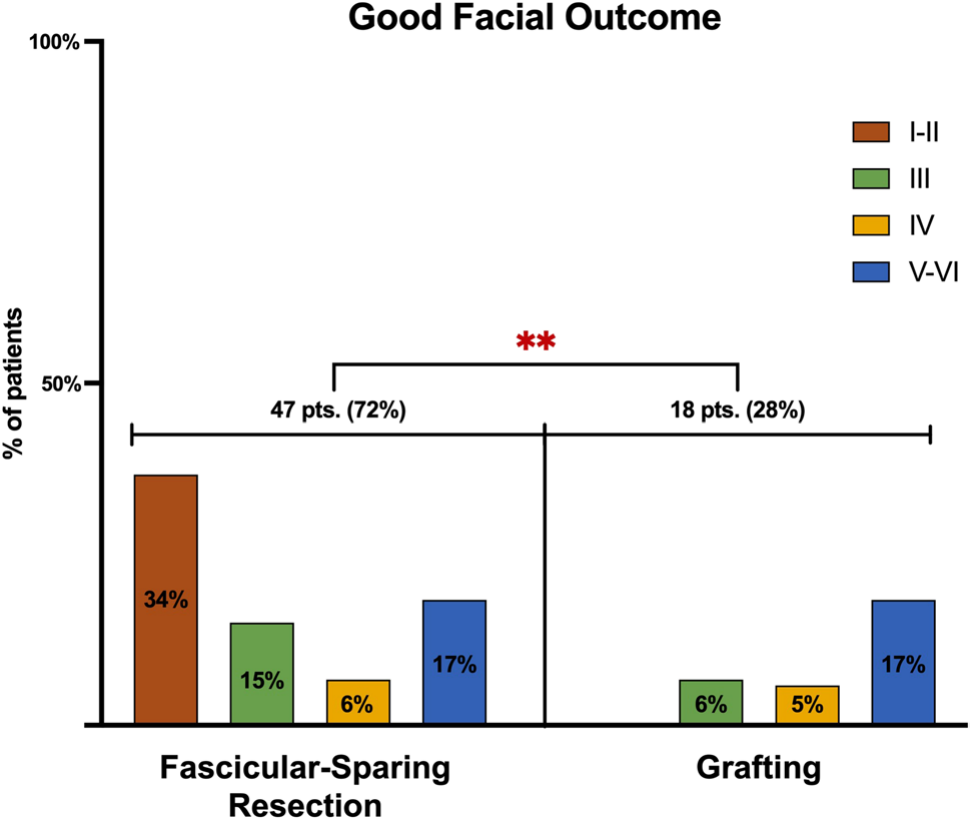
Bar graph showing the statistical comparison between the efficacy of each technique in preserving the facial nerve function in terms of good facial outcomes, set at HB grade III. Pts patients; **p < 0.05

In the grafting group, a good outcome was achieved in 28% (18) of patients, of which 17% (9) have an HB V–VI at admission (Fig. 6).

### Illustrative case

**Fig. 7:**
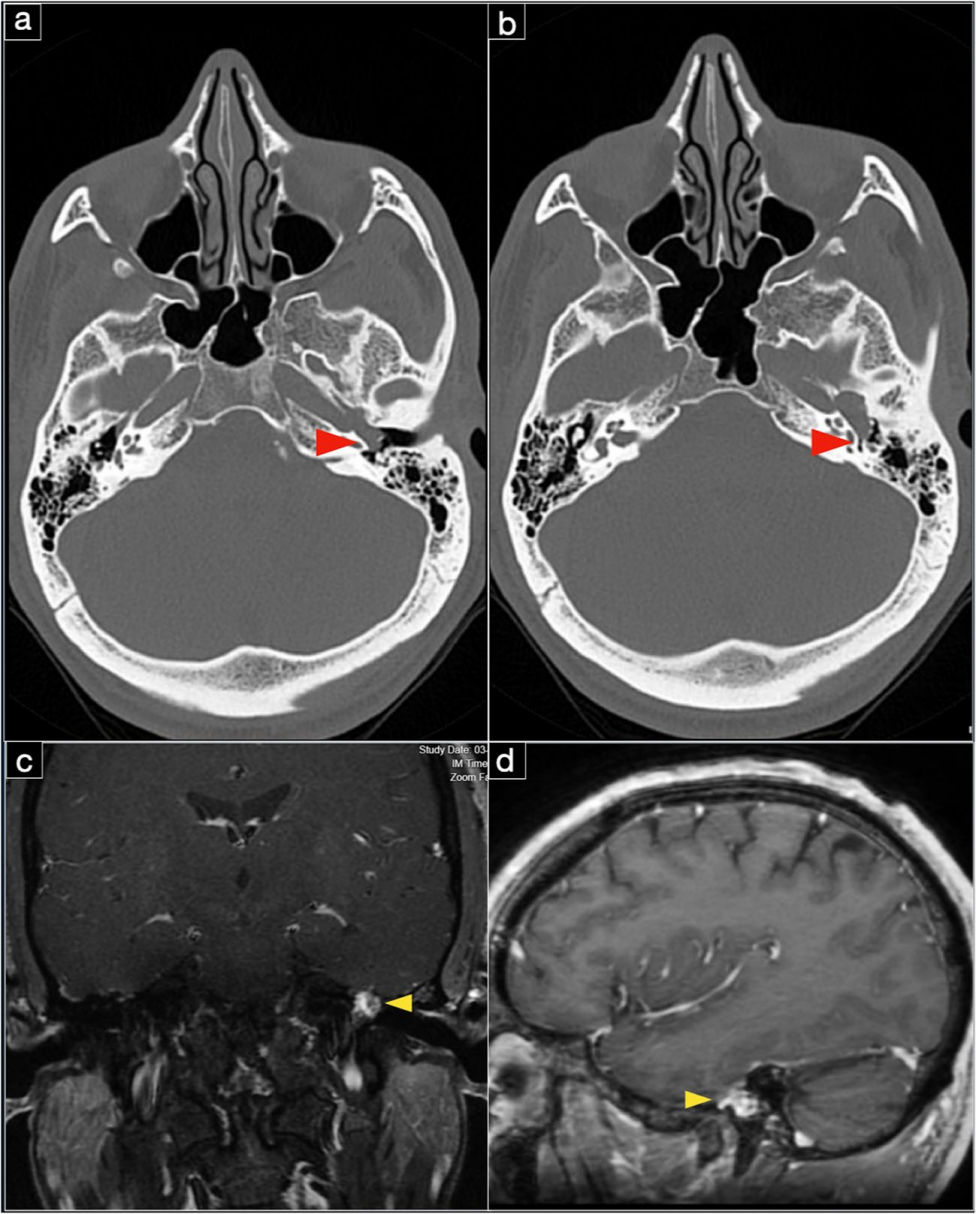
CT scan bony window in the axial plane (**a**, **b**) demonstrating the enlargement at the level of the intralabyrinthine segment with the protrusion into the middle ear (red arrowheads). Preoperative gadolinium contrast-enhanced T1-weighted MRI in coronal (**c**) and sagittal (**d**) plane demonstrating the enhancing (yellow arrowheads)

A 38-year-old, otherwise healthy man presented with a history of progressive left facial weakness, slight facial asymmetry, and left ear fullness over 9 years. His symptoms worsened in recent months with an increased effort required to close the ipsilateral eye. The family history was unremarkable. The neurological examination revealed a left facial palsy HB grade III, left-sided reduced hearing, and the left dry eye closable with effort. Audiometric testing demonstrated a conductive hearing loss in the left ear. A CT scan revealed the enlargement at the level of the labyrinthine segment with a tumor protruding into the middle ear (Fig. 7a, b). On MRI, the lesion was densely enhancing, involved the left geniculate ganglion, and abutted the intrapetrous segment of the left internal carotid artery (Fig. 7c, d). Radiographically, the lesion was initially diagnosed as a facial nerve schwannoma.

The patient underwent a zygomatic extended middle fossa approach.

**Fig. 8:**
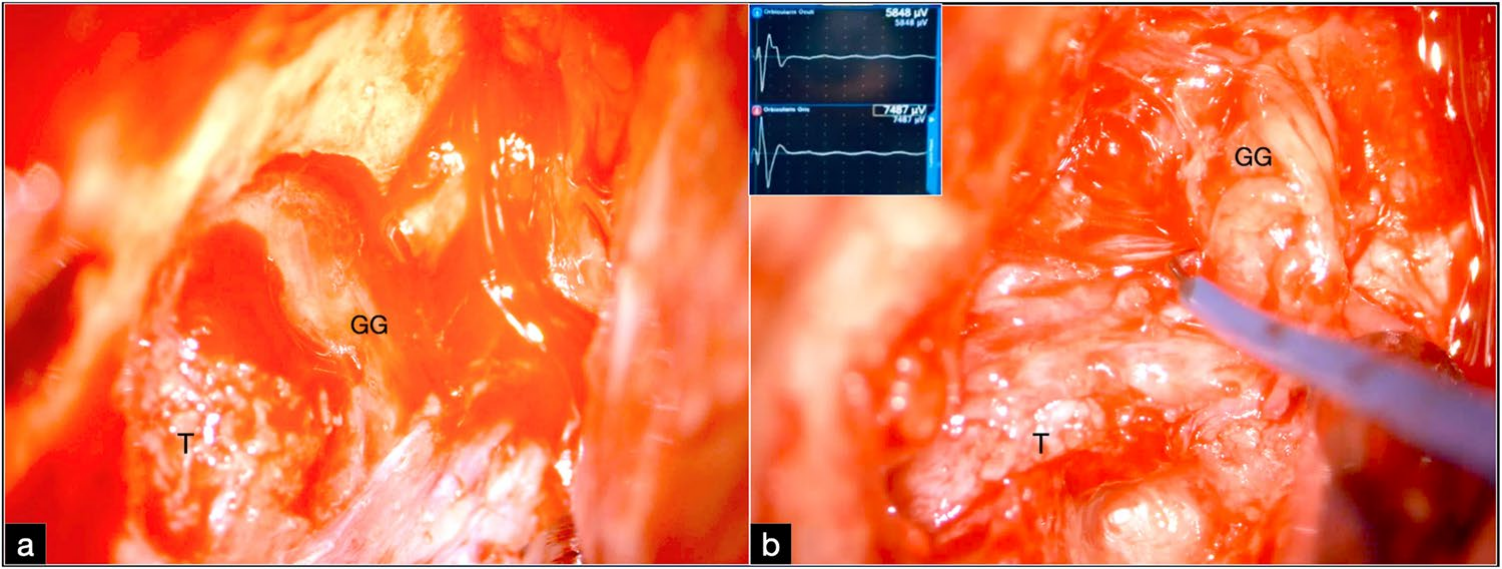
**a** Exposure of the left middle fossa and identification of the tumor and the geniculate ganglion. **b** Fascicular-sparing resection of the tumor by means of fine stimulating dissector. Insets in **b**: electromyography of the facial nerve. Stimulation at a threshold of 0.2 mA. GG geniculate ganglion, T tumor

Neuronavigation and neurophysiological monitoring were used. The latter included the seventh, third, fifth, and sixth cranial nerves. Brainstem auditory and somatosensory evoked potentials were also monitored. The patient was placed supine with the head elevated at 20° and rotated at 45° to the contralateral side. A preauricular skin incision, curving back over the ear, was performed. The zygoma was cut and mobilized caudally, leaving the attachment of the masseteric muscle. A temporal craniotomy was performed with the resection of the squama until the floor. Through the extradural dissection of the middle cranial fossa, a soft, reddish mass was revealed at the level of the facial hiatus (Fig. 8a). Additional drilling was performed in the dehiscent bone and carried out to the roof of the internal auditory meatus to achieve a total exposure of the tumor. The area of the entry into the tumor was identified by means of negative stimulation, and a gross total fascicle-sparing resection was performed. The tumor was progressively debulked with an ultrasonic aspirator from the inside to the periphery. A nerve stimulation of 0.2 mA was useful to identify and preserve the facial nerve fascicles, with the nerve continuing to stimulate at 0.2 mA at the end of the resection [19] (Fig. 8b).

**Fig. 9:**
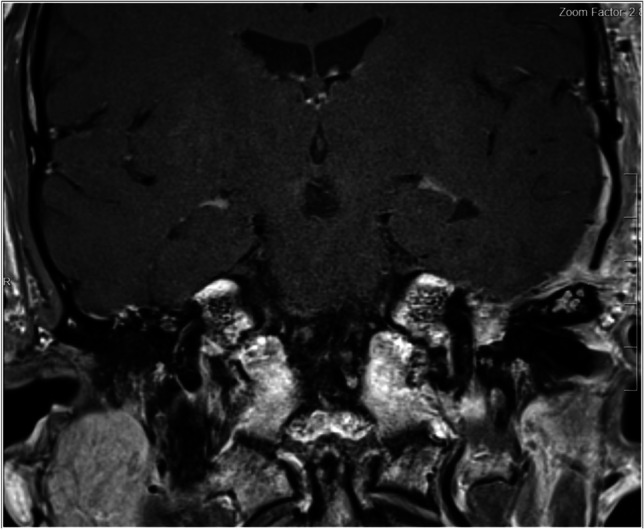
Postoperative gadolinium contrast-enhanced T1-weighted MRI in the coronal plane

**Fig. 10:**
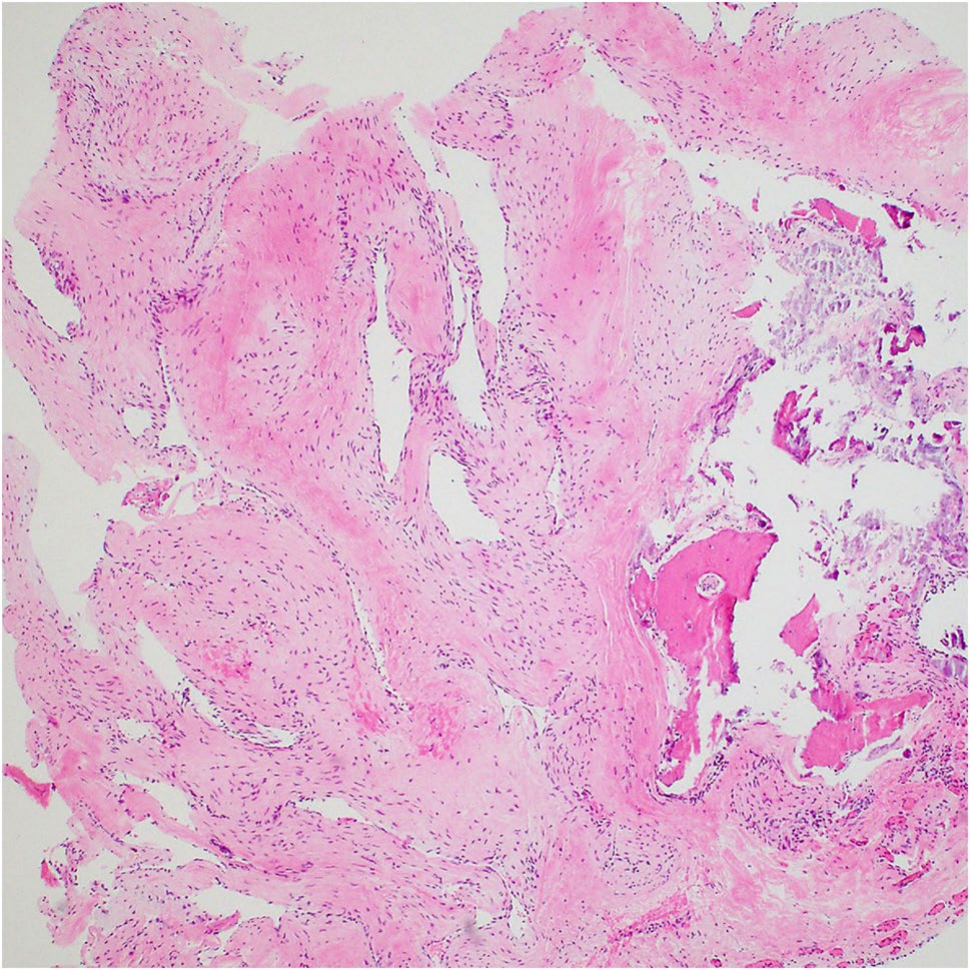
Photomicrograph of the intraosseous lesion exhibiting fibrotic stroma and large, dilated, thin-walled vessels characteristic of hemangioma

The postoperative course was uneventful, and the facial deficit remained stable. Postoperative MRI confirmed the total resection of the tumor (Fig. 9), and the postoperative hearing test was stable. Pathology was conclusive of a GGH S-100 negative (Fig. 10).

Artificial tears and lubricant for the left eye were given at discharge on the second postoperative day. A plastic surgeon treated facial palsy with eyelid weight placement. The patient referred the symptoms subjectively improved over the following months.

## Discussion

### Comparison of surgical options

GGHs are uncommon in neurosurgical practice and, accordingly, underestimated in the literature. An early clinical diagnosis is difficult to achieve, and the management is controversial.

Incidental diagnosis, paucisymptomatic patients, or recent-onset symptoms should be candidates for the wait-and-see approach, carried out by seriate imaging follow-up.

Pieces of evidence support prompt surgical intervention since it proved the best treatment option for more symptomatic cases. Whenever possible, it is recommended in patients with an HB grade ≥ III [8, 10, 15, 20, 21].

Surgery of GHHs has two goals, namely, tumor resection and preservation of the nerve function. The approach is selected based on the tumor extension and preoperative hearing assessment. The middle fossa approach is the best choice to maximize the extent of resection, as stressed by different groups [4, 5, 7, 9–14, 16, 20, 22–31]. The zygomatic osteotomy widens the surgical corridor and illuminates the blind spots in the depth of the surgical field, while the drilling of the roof of the internal auditory meatus allows for achieving the total exposure of the tumor [32–36]. The middle fossa approach enables the early identification of the facial nerve during the microdissection, facilitating the preservation of nerve function. Furthermore, it allows for greater bony removal and the peripheric decompression of the distal portion of the nerve.

In 2016, Lahlou and colleagues presented the results of a series of 10 GHHs affected by severe facial palsy (HB V–VI) treated with a middle fossa approach. A gross total resection of the tumor was achieved in 100% of cases, and the postoperative HB grade improved in 94% of them [21].

Regarding the intraoperative technique, two different strategies were described: the fascicular-sparing removal of the tumor and the intentional interruption of the nerve followed by grafting. Since the intense perineural reaction results in intimate adhesion of the tumor to the facial nerve, an accidental break may occur, and a repair may be required. The facial nerve rerouting is performed via end-to-end anastomosis or grafting with greater auricular or sural nerve [11, 15, 20, 21, 28].

Our critical appraisal of the literature revealed the superiority of the fascicular-preserving technique in saving nerve integrity and reaching better outcomes [3, 4, 9, 12, 14, 22, 25, 29, 30]. Indeed, 64% of patients with an improved/unchanged facial outcome and 72% with a postoperative HB grade III were treated via a fascicular-sparing resection of the tumor.

This technique permitted the maintenance of facial integrity, especially for patients who presented with a preserved facial function (I–II HB). The grafting technique demonstrated only slight improvements, limited to patients with V–VI grades at admission.

In accordance with our case, the analysis proved the fascicular-preserving technique more suitable for patients presenting with a full or partially preserved facial function (I–III). As a matter of fact, almost all patients with I–III HB improved or remained stable; contrariwise, none of those in the graft group reported an improvement in facial palsy [3–5, 8–12, 14, 15, 20–25, 29, 30].

These results are critical since the maintenance of eye closure function (HB I–III) significantly affects the patient’s quality of life.

Despite the grafting giving a chance of recovery in V–VI HB cases, saving the nerve fibers through a fascicular-sparing resection is always advisable.

### Timing of surgical intervention

In regard to the surgical timing, the rationale for a prompt resection at the earliest symptom lies in a higher probability of preserving the facial nerve function. As reported by Oldenburg et al., facial nerve weakness is the typical onset of GGHs, found in 94% of cases, followed by sudden onset of facial spasms [15]. The succeeding middle ear invasion may result in sensorineural or conductive hearing loss, vertigo, and otalgia.

GGHs cause facial nerve dysfunction at the early stages of growth due to direct compression and ischemia of the facial nerve [37, 38]. In 2014, Wang and colleagues presented a series of 16 surgical cases of GGHs, all with facial palsy [20]. They demonstrated a significant difference in the preservation rate of facial nerve function, which was 20% and 83.3% (*p* < 0.05) in patients with long- and short-lasting deficits, respectively.

Similar to a previous report by Sataloff et al. for facial nerve schwannomas [39], facial function and the overall outcome are strictly related to the integrity of the endoneurium. Moreover, an early resection, within the first year of onset, is recommended due to progressive tumor growth invading the space between nerve fascicles resulting in irreversible damage to the nerve with subsequent loss of facial motor end plates [8, 15, 40].

### Characteristics of GGH

Based on the clinical onset, GGHs are frequently misdiagnosed for schwannomas or meningiomas. Precise radiographic and clinical preoperative identification is critical to direct proper clinical management. The high-resolution neuroimaging techniques are helpful for proper identification and differential diagnosis. While not recognizing the small GGHs at the early stages of growth, CT discerns the so-called “osseous” types, which appear as irregular osteolytic lesions. Osseous GGHs harbor intralesional calcification and have a sunburst or “honeycomb” radiographic appearance [41], while facial nerve schwannomas present as focal expansions with well-defined borders [9, 17, 42]. MRI diagnostic characteristics are iso- and hyperintense on T1- and T2-weighted images, respectively [43].

Histopathology is pivotal in the diagnosis of hemangioma since hematoxylin and eosin stain GGHs show numerous enlarged vascular channels interspersed with intralesional lamellar bony trabeculae mainly in the bone variant [41]. Immunohistochemical profiling may provide additional information. The lack of the S-100 is decisive, as it was in our case [44].

## Conclusion

GGHs are rare vascular tumors. They cause progressive facial nerve dysfunction from the early stages of growth. Long-lasting facial nerve palsy is related to a worse outcome, and a timely and accurate diagnosis is essential.

GGHs are treated by surgical resection, which should be performed within the first year of symptomatic onset. The fascicular-sparing technique, through the zygomatic middle fossa approach, is effective in preserving facial nerve integrity. It is advisable mainly for patients who presented with a full or partially preserved facial function, intended as HB grades I–III.

Conversely, the grafting technique proved suitable for patients with severe facial dysfunction at admission (HB IV–VI). The protection of nerve fascicles during dissection is critical in achieving the best outcome and especially preserving the eye closure, which proved to be the main symptom driving the patient’s quality of life.

## Data Availability

All data are included in the main text.
